# Incidence and risk factors of urinary incontinence in women visiting Family Health Centers

**DOI:** 10.1186/s40064-016-2965-z

**Published:** 2016-08-11

**Authors:** Meral Kılıç

**Affiliations:** Department of Midwifery, Faculty of Health Science, Ataturk University, Erzurum, Turkey

**Keywords:** Urinary incontinence, Prevalence, Risk factors

## Abstract

**Background:**

The objective of this study is to determine the incidence and the risk factors of the urinary incontinence in women visiting the Health Family Center.

**Methods:**

430 women, who visited three Family Health Centers in the city center of Erzurum for any reason between 25 November and 20 January 2016, were included in this study without any sampling. The data were collected by using the face-to-face interview method. Percentage distribution, Chi square test, and logistic regression analysis were used in order to analyze the data.

**Results:**

It was determined that 37.2 % of these women had urinary incontinence, but only 29.3 % of them visited a physician because of this complaint. Among a total of 160 women with urinary incontinence findings, stress type incontinence was observed at the highest rate (33.7 %), which was followed by mixed type (31.8 %), urge type (20.6 %) and other types (overflow, continuous urinary incontinence) (13.7 %). It was found that urinary incontinence had a significant correlation with the number of children, genital prolapse, duration of delivery longer than 24 h, diabetes and urogenital infection, but not with the age at the first and last childbirth, presence of the episiotomy, birth weight over 4 kg, and smoking.

**Conclusions:**

It was determined that one-third of the women had urinary incontinence and certain medical and obstetric conditions were affecting the development of urinary incontinence. It is thought that it is important for the healthcare personnel to take the progression of the urinary incontinence under control by preventing the risk factors and to encourage the patients to seek treatment with the help of the proper information indicating that urinary incontinence is a treatable and preventable condition.

## Background

Urinary incontinence (UI) is a storage symptom and defined as the complaint of any involuntary loss of urine (Abrams et al. 2010). Worldwide over 200 million people have an incontinence problem, which is encountered often in healthy persons, especially in women. Its prevalence is between 15 and 50 % (Basak et al. 2013; Norton and Brubaker 2006; Nygaard et al. 1994). Although urinary incontinence is not a life-threatening problem, it may cause emotional disorders like depression due to continuous wetness and irritation. In the literature, it is shown that women with incontinence symptoms are more prone to depression, have higher anxiety levels, and feel more humiliated compared to women without incontinence symptoms (Berglund et al. 1994; Valvanne et al. 1996; Williams 2004). Due to the fear of incontinence, routine daily life activities might be stressful and embarrassing. It is determined that the incontinence might affect negatively traveling, shopping, playing with children, exercising and sexual activity and the quality of life might impair (Williams et al. 2001).

Urinary incontinence may develop as a result of aging and childbirth. Although it is common among elderly people, it should not be interpreted as a geriatric disorder since it is not rare among middle-aged and young people (Ostergard and Bent 1996). In a study, the prevalence of UI was 12.8 % in young people, 36.1 % in middle-aged people, and 35 % in old people (Chiarelli et al. 1999). In another study, it was shown that UI was more common in young women rather than 60 year-old people (Nygaard et al. 1994). One study reported that the highest rate of stress incontinence was encountered in women aged between 25 and 49 years (Hannestad et al. 2000).

In women, the most significant risk factors associated with UI are pregnancy, childbirth, age, body mass index, and previous hysterectomy. Gender, previous pelvic surgery, obesity, chronic cough, depression, type of childbirth, smoking, genetics, menopause, hormone replacement therapy in the post-menopausal period are the other risk factors (Doughty 2012; Melville et al. 2005; Ortiz 2004). The most apparent risk factors among women with UI have been reported to be vaginal delivery, macrosomia, high number of pregnancies, interventional births, and vaginal episiotomy (Kocaöz and Eroglu 2002; Kasikçi et al. 2015).

In Turkey, most of the women are multiparous; therefore, they have urinary incontinence as a result of the weakened pelvic muscles, which affect them seriously in medical, physical, social, psychological and economic aspects and impair their quality of life (Bates et al. 1979; Taşkın 2007). The context of the concept of quality of life is closely related with nursing and midwifery interventions. Therefore, the activities of nurses and midwives should focus on behaviors and reactions, which have a positive effect on quality of life (Kızılkaya 2002; Şirin and Kavlak 2008). Within this scope, prevention of the development of the incontinence and having an active role in the treatment of women with this disorder are one of the basic responsibilities of the nurses and midwives. In order to prevent urinary incontinence, risk factors should be well known (Bates et al. 1979). This study was conducted in order to determine the incidence and risk factors of the urinary incontinence among women applying to the Family Health Center.

## Participants and methods

### Participants

This study was conducted in Filiz Dolunay, Yenişehir, and Yıldızkent Family Health Centers in the city center of Erzurum between 25 November 2015 and 20 January 2016. The sample group consisted of 430 women visiting these three Family Health Centers in the city-center of Erzurum for any reason. All women were included in the study without any sampling. The inclusion criteria were as follows; being over 20 years of age, presence of previous childbirth, willingness to participate, and lack of any acute, mental, and auditorial disorder.

### Data collection

The “Information Form” prepared by the researchers was used to record the data. The Personal Information Form was prepared according to the samples in the literature (AnChiu et al. 2008; Karaçam and Eroğlu 2003; Milsom et al. 1993; Ortiz 2004). There was a total of 22 questions in this Personal Information Form regarding age, educational background, socio-demographic, medical and obstetric characteristics, UI experience, UI frequency, and urine volume. The patients who answered any of the data marked with (*) in Table 2 as “yes” were accepted to have “urinary incontinence findings” in accordance with the literature (Table 2). In the section where we aimed to determine the severity level of UI, the patients were asked questions regarding frequency of urinary incontinence and whether or not they use pad. Those who had one or more incidence of incontinence and/or those who did not use pad were classified as “mild UI”. Those who had incontinence problem almost every day and had to use continuously pad were accepted as “severe UI”. The cases between these two categories, the cases who had incontinence problem more than two per a week though not every day and for whom pad usage was not consistent, were called as “moderate UI” (Table 2).

Those, having history of any chronic disease (diabetes mellitus, pelvic organ prolapse (POP), repeated urinary tract infection etc.,) diagnosed by the physician previously, were accepted to have “urinary incontinence findings”.

Those having urinary tract infections which progressed with three and more number of symptomatic attacks within the last 1 year and were diagnosed by the physician were accepted to have “repeated urinary tract infection”.

The data were collected by using the face-to-face interview method from the women who visited the mentioned Family Health Centers for any reason or were reached at home as from November 2015. Each interview lasted for 10–15 min.

### Statistical analysis

Encoding and assessment of the data were performed by using SPSS v20.0 software (Statistical Package for Social Science). Number and percentage distributions were used for descriptive statistics and the related evaluations were performed by using Chi square test and logistic regression analysis.

### Drawbacks and limitations of the study

The memory impairment of the elderly people may raise difficulties concerning the retrospective information especially regarding childbirth. However, the planned study was important for understanding the etiology and risk factors of UI.

### Ethical issues

In order to conduct this study, ethical approval of Atatürk University Institutional of Health Sciences Review Board according to the Declaration of Helsinki (IRB No. CMUH-34/21.12.2015) was received. All participants gave informed written consent prior to inclusion in the study. As the questions had to be answered voluntarily, we informed that they were free in participating in the study and we assured their willingness and voluntariness. After the participants were informed and their questions were answered about the study, their verbal and written consent were obtained before starting the study. They were also informed that they could withdraw from the study anytime.

## Results

36.3 % of the women were aged between 30 and 39 years. 48.4 % of them were primary school graduates, 97.9 % were married, and 72.2 % did not have a job (Table 1).

**Table 1:** Maternal characteristics (n = 430)

	Number	%
Maternal age		
20–29	87	20.2
30–39	156	36.3
40–49	120	27.9
50 and above	67	15.6
Educational status		
Illiterate	44	10.2
Primary school graduate	208	48.4
Secondary school graduate	178	41.4
Married		
Yes	421	97.9
No	9	2.1
Employment status		
Yes	119	27.8
No	311	72.2

12.5 % of the women participating in the study were observed to have urinary incontinence during coughing, sneezing or daily activities (stress type), 11.8 % during coughing, sneezing and when in need of urinating (mixed type), 7.67 % when in need of urinating (urge type), and 5.11 % in cases of other types (reflex type UI developing due to congenital or subsequent anatomic abnormalities such as overflow incontinence, urethrovaginal fistulas, ectopic ureter) (Table 2).

**Table 2:** Characteristics related to urinary incontinence findings (n = 430)

	Number	%
Characteristics		
Urinary incontinence during coughing, sneezing or daily activities (Stress type)*	54	12.5
Urinary incontinence during coughing, sneezing and when in need of urinating (mixed type)*	51	11.8
Urinary incontinence when in need of urinating (urge type)*	33	7.67
Any case of urinary incontinence	160	37.2
Severity of urinary incontinence		
Those who had once or less incidences of incontinence per week or/and did not use pad (mild UI)	124	28.8
The cases who had incontinence problem more than two per a week though not every day and for whom pad usage was not consistent, (moderate UI)	26	6.04
Those who had incontinence problem almost every day and had to use pad (severe UI)	10	2.32

*: (*) patients who answered any of the data marked with (*) as “Yes” were accepted to have “urinary incontinence findings” in accordance with the literature

The patients who answered any of the data marked with (*) in Table 2 as “yes” were accepted to have “urinary incontinence findings” in accordance with the literature. Accordingly, 160 (37.2 %) of a total of 430 patients participating in the study were determined to have any urinary incontinence (Table 2).

It was determined that only 29.3 % of these patients applied a healthcare organization due to urinary incontinence and UI findings were experienced between 1 and 12 months at higher rate (Table 3). 33.7 % of the women were observed to have urinary incontinence during coughing, sneezing or daily activities (stress type), 20.6 % during coughing, sneezing and when in need of urinating (mixed type), 31.8 % when in need of urinating (urge type), and 13.7 % in cases of other types (reflex type UI developing due to congenital or subsequent anatomic abnormalities such as overflow incontinence, urethrovaginal fistulas, ectopic ureter) (Table 3). In the studies determining severity of UI by evaluating frequency of UI and the usage of pad, the results obtained for all the sample group were determined as 77.5, 16.3, and 6.2 % for mild, moderate and severe UI (Table 3).

**Table 3:** The Distribution of the patients with urinary incontinence findings based on urinary incontinence types

	Number	%
Types (n = 160)		
Stress type urinary incontinence	54	33.7
Urge type urinary incontinence	33	20.6
Mixed type urinary incontinence	51	31.8
Others	22	13.7
Urinary incontinence (n = 430)		
Yes	160	37.2
No	270	62.8
Applying to a health organization due to urinary incontinence (n = 160)		
Yes	47	29.3
No	113	70.2
Period of experiencing involuntary leakage of urine (n = 160)		
1–12 months	105	65.6
13–48 months (4 years)	26	16.2
49–96 months (8 years)	9	5.6
97 months	20	12.6
Severity of urinary incontinence (n = 160)		
Those with mild urinary incontinence	124	77.5
Those with moderate urinary incontinence	26	16.3
Those with severe urinary incontinence	10	6.2

42.2 % of them gave their first birth between the ages of 20–25 years, age of 34.5 % at the last childbirth was between the ages of 30–34 years, and 52.9 % had 1–2 children. 30 % had a duration of delivery longer than 24 h, 43.2 % had an episiotomy, and 34.7 % gave birth to a child with a birth weight over 4 kg. It was found determined that 15.3 % of the women had genital prolapse, 21.9 % had diabetes, 8.8 % had a urogenital infection, and 34 % were smokers (Table 4).

**Table 4:** The distribution of obstetric characteristics of women (n = 430)

	Number	%
Age at the first childbirth		
20 and below	178	41.5
20–25	182	42.2
25 and above	70	16.3
Age at the last childbirth		
25 and below	62	14.6
25–29	118	27.8
30–34	152	34.5
35 and above	98	23.1
Number of children		
1–2	228	52.9
3–4	142	33.1
5 and above	60	14.0
Duration of delivery longer 24 h		
Yes	129	30.1
No	301	69.9
Presence of episiotomy		
Yes	186	43.2
No	244	56.8
Birth weight over 4 kg		
Yes	149	34.7
No	281	65.3

It was determined that 15.3 % of the women had POP, 21.9 % had diabetics, 8.8 % had urogenital infection, and 34 % were smokers (Table 5).

**Table 5:** The distribution of urinary incontinence-related characteristics of the women

	Number	%
Genital prolapse	n = 430	
Yes	66	15.3
No	364	84.7
Diabetes	N = 430	
Yes	94	21.9
No	336	78.1
Urogenital infection	n = 430	
Yes	38	8.8
No	391	91.2
Smoking	n = 430	
Yes	146	34.0
No	284	66.0

According to the correlation of UI with medical and obstetric characteristics and to the logistic regression analysis of the risk factors; number of children, genital prolapse, duration of delivery longer 24 h, diabetes, and urogenital infection had a significant correlation with UI, but not with age at the first and last childbirth, presence of episiotomy, birth weight over 4 kg, and smoking. (Table 6).

**Table 6:** Logistic regression analysis regarding the correlation of the urinary incontinence and medical and obstetric characteristics

	Urinary incontinence (UI)		Test value and p	OR
	With UI number	Without UI number (%)		
Age at the first childbirth				
20 and below	71 (39.9)	107 (60.1)	_χ_^2^ = 4.766	1.036 (752–1.426)
20–25	68 (38.2)	110 (61.8)	p = .190	p = .830
25 and above	21 (28.3)	53 (71.7)		
Age at the last childbirth				
25 and below	23 (37.1)	39 (62.9)	_χ_^2^ = 4.093	1.144 (.886–1.512)
25–29	35 (29.7)	83 (70.3)	p = .252	p = .342
30–34	58 (39.5)	89 (60.5)		
35 and above	44 (42.7)	59 (57.2)		
Number of children				
1–2	66 (29.1)	162 (70.9)	_χ_^2^ = .196	.619 (.424–.904)
3–4	61 (43.0)	81 (57.0)	*p* *=* *.000*	*p* *=* *.013*
5 and above	33 (55.0)	27 (45.0)		
Duration of delivery longer 24 h				
Yes	58 (45.0)	71 (55.0)	_χ_^2^ = 4.826	1.694 (1.067–2.692)
No	102 (33.8)	200 (66.2)	p = .028	*p* *=* *.026*
Presence of episiotomy				
Yes	73 (39.2)	113 (60.8)	_χ_^2^ = .524	995 (911–1.087)
No	87 (35.7)	157 (64.3)	p = .769	p = 909
Birth weight over 4 kg				
Yes	51 (34.2)	98 (65.8)	_χ_^2^ = .919	655 (412–1.040)
No	109 (38.9)	172 (34.2)	p = .338	p = .073
Genital prolapse				
Yes	42 (63.6)	24 (36.4)	_χ_^2^ = 23.306	2.878 (1.597–5.185)
No	118 (32.4)	246 (67.6)	*p* *=* *.000*	*p* *=* *.000*
Diabetes				
Yes	34 (36.2)	60 (63.8)	_χ_^2^ = 16.548	984 (792–1.221)
No	126 (37.6)	209 (62.4)	*p* *=* *.000*	p = .881
Urogenital infection				
Yes	23 (60.5)	15 (39.5)	_χ_^2^ = 9.700	1.899 (895–4.030)
No	137 (34.9)	255 (65.1)	*p* *=* *.002*	p = .095
Smoking				
Yes	52 (35.6)	94 (64.4)	_χ_^2^ = .240	1.061 (672–1.675)
No	108 (38.0)	176 (62.0)	p = .624	p = 801

Significant values are in italics

In the present study, according to the logistic regression analysis regarding the relation of the urinary incontinence and medical and obstetric characteristics; it was determined that number of children, long duration of delivery, and POP were risk factors for UI in women. It was also found that the age at the first and last childbirth, presence of episiotomy, birth weight over 4 kg, diabetes, urogenital infection, and smoking were not risk factors for UI (Table 6).

## Discussion

Urinary incontinence is an important medical and social public-health problem for people with incontinence, his/her family and due to its cost to the healthcare system (Shakhatreh 2005). In Turkey, the prevalence of UI vary between 15 and 50 % (Basak et al. 2013; Bilgili et al. 2008). In their study Hunskaar et al. (2003) determined that the prevalence of UI among women in European countries was 23 % in Spain, 41 % in Germany, 42 % in the UK, and 44 % in France. In the present study, it was found that 37.2 % of the women had urinary incontinence. Similar to results of the present study, Kök et al. (2006) reported in their study that 37.11 % of the women visiting the gynecology outpatient clinic had urinary incontinence. In the study conducted by Ateşkan et al. (2000), the incidence of UI was 39.7 % in the sample group. The difference in its prevalence might be a result of different ages of patients included in the study, the different diagnostic criteria used for UI, different survey forms, and different data collection methods (Basak et al. 2013).

It was found that although one-third of the women had urine leakage problem, 70.7 % did not visit a physician for this complaint. The ratio of the patients visiting a physician for urinary incontinence was 14 % in the study of Hägglund et al. (2001), 38 % in the study of Kinchen et al. (2003), and 27 % in the study of Yu et al. (2003). In the literature, the main reasons reported for not visiting a physician were the hope for recovery of symptoms, shyness and hesitation to talk with a physician about this problem, fear of operation and the assumption that it is a natural consequence of childbirth and aging (Kinchen et al. 2003; Ortiz 2004). The severity of the incontinence is also affecting the rate of visiting a physician. Another evidence directly demonstrates that perceived UI severity is a predictor of help-seeking behavior among incontinent women (Koch 2006). Another study revealed that lower subjective UI severity or lower perceived UI severity contributed to longer delay to treatment (Wu et al. 2015). In the present study, it was determined that 77.5 % of the women had mild urinary incontinence, 16.3 % moderate urinary incontinence, and 6.2 % severe urinary incontinence. Therefore, the reason for the low rate of visiting a physician may be the fact that majority of the women had mild UI. This may delay the diagnosis and treatment of the disorder (Kinchen et al. 2003). For this reason, the awareness about the importance of the early diagnosis and treatment in UI should be increased. People, who have incontinence but maintain apparently a life without much disturbed by this condition, may be encouraged to seek health advice by informing them that this disorder is treatable and preventable (Kızılkaya 2002).

SUI was the predominant subtype among women in the present study. SUI was the commonest subtype of UI in many studies conducted on females (Çiftçi and Günay 2011; Ghafouri et al. 2014; Altaweel and Alharbi 2012; Al-Badr et al. 2012). However, other studies reported mixed UI as being more common than stress UI (El-Azab et al. 2007; Rizk et al. 2001).

Childbearing may cause urinary incontinence in women. The present study revealed a significant correlation between the number of children and UI. In a previous study, it was reported that UI was 5.5 % among nulliparous women, 10.6 % among primiparous women, and 16.4 % among women with more than 3 deliveries (Milsom et al. 1993). In another study, it was showed that the prevalence of both stress and urge incontinence was lower among nulliparous women and higher among women with 5–6 deliveries (Shakhatreh 2005). Kocaöz and Eroglu (2002) found a significant correlation between stress incontinence and several risk factors such as the number of pregnancies, miscarriages, number of births, number of miscarriages, birth location, and complaint of UI during pregnancy. This may be associated with the impairment in the pelvic muscle nerves during birth, the development of atrophy in muscles, and the development of prolapse over time (Kasikçi et al. 2015).

In the present study, it was found that there was a significant correlation between the UI and delivery lasting longer than 24 h and the logistic regression analysis revealed that delivery lasting longer than 24 h was a risk factor for UI. However in a previous study, it was reported that delivery lasting longer than 24 h did not have a prominent effect on UI (Thom and Brown 1998). Another study revealed that delivery lasting longer than 24 h increased the development rate of moderate or severe urinary incontinence 1.3 times (Rortveit et al. 2003). It was suggested that the pudendal nerve damage after long and difficult delivery and pubococcygeus muscle weakness were possibly the reason for urine leakage. It is thought that the mechanism of the nerve damage during the delivery may be both the stretching of the pelvic floor and direct pressure of the fetal head on the nerve along the pelvic side walls (Thom and Brown 1998).

Another factor affecting the development of UI is the childbirth with a birth weight over 4 kg. Its probable consequence is the damage to the pudendal nerve, connective tissue and pelvic floor muscles (Højberg et al. 1999). In the present study, it was determined that history of a big baby was not a risk factor for UI. Several studies showed that the childbearing with a birth weight over 4 kg did not cause the development of UI (Connolly et al. 2007; Viktrup et al. 1992). On the other hand, in some studies, it was found that childbearing with a birth weight over 4 kg affected UI (Højberg et al. 1999; Oliveira et al. 2010).

In the present study, it was found that episiotomy did not affect the development of UI. Bilgili et al. (2008) found no significant correlation between vaginal episiotomy, age at first childbirth, and UI. Other studies also showed that vaginal episiotomy had no effect on UI (Karaçam and Eroglu 2003; Sartore et al. 2004). In a previous study, it was found that regarding the urinary incontinence in the 3rd month after the delivery, the score/status of urinary incontinence was significantly higher among women with episiotomy compared to women without episiotomy (Chang et al. 2011). According to the literature, it was concluded that episiotomy was effective in the prevention of the anterior perineal laceration, but not in the prevention of the perineal damage, and especially in the prevention of urinary and anal incontinence and pelvic floor relaxation (Hartmann et al. 2005).

Genital prolapse and urinary incontinence might be seen concomitantly in the same patient. They might cause cystocele and micturition difficulties (Kızılkaya 2002). The present study revealed a significant correlation between UI and prolapses. In a previous study, it was determined that among women aged over 65 years, the risk of urinary incontinence was higher in women with a genital prolapse compared to women without a genital prolapse (Bilgili et al. 2008). It is thought that the damage to the nerves and muscles in the pelvic floor is the main causative factor regarding the development of the POP. Therefore, if the genital prolapse is developed, urinary and anal incontinence might develop in the same patient (Meschia et al. 2002).

The present study revealed a correlation between urinary infection and UI. In studies with similar results, it was shown that there was a significant correlation between the history of frequent urinary tract infections and the incidence of urinary incontinence (Hägglund et al. 1999; Isikli et al. 2011). The reason for this might be the increase in detrusor muscle contractions and sphincter pressure depending on the infection (Basak et al. 2013).

Another factor affecting urinary incontinence is diabetes mellitus. In the present study, it was determined that diabetes affected the development of UI. Similar to results of the present study, some other studies revealed a significant correlation between diabetes and UI (Brown et al. 1996; Nakayama et al. 1997; Shakhatreh 2005). On the other hand, three other studies did not identify diabetes as a significant risk factor for urinary incontinence (Melville et al. 2005; Song et al. 2005; Zhu et al. 2008). Diabetes mellitus has a remarkable role on urinary incontinence, as a result of glycosuria, overactivity of the detrusor muscle, recurrent urinary infections, and diabetic cystopathy (Basak et al. 2013). It was stated that hyperglycemia might cause incontinence if it increases urine volume and damages the nerve conduction of the bladder (Brown et al. 1996).

The present study revealed no significant correlation between the ages at the first and last childbirth and UI. Similar to results of the present study, Erata et al. (2002) reported that there was a negative correlation between the age at the first childbirth and UI and no correlation between the age at the last childbirth and UI. Persson et al. (2000) indicated that there was no correlation between the age at the last childbirth and UI but there was a positive correlation between the age at the first childbirth and stress incontinence. This result was interpreted as the fact that the pelvic floor muscles of young nulliparous women were more sensitive and could be affected more (Persson et al. 2000).

Masue et al. (2010) reported that women aged over 28 years at their first childbirth had 1.9 increased odds of SUI compared to women aged 25 years or younger at their first childbirth. Possible reasons for this association are that increased maternal age is a risk factor for intrapartum pelvic floor trauma and tissue recovery of the pelvic floor may slow with age; however, there is no scientific evidence to support this view (Masue et al. 2010).

The present study and other studies could not reveal any correlation between UI and smoking habits (Erata et al. 2002; Højberg et al. 1999). Sampselle et al. (2002) conducted a case–control study on 606 women and found that the risk of the urinary incontinence among smoking women was 2.5 times higher. In other studies, it was showed that smoking and being in the postpartum 12th month were risk factors for urinary incontinence (Burgio et al. 2003; Miller et al. 2003). Regarding the correlation smoking and leakage of urine, there are rational explanations concerning sphincteric, neurological and anatomic mechanisms. The adverse effect of smoking depends on estrogen levels, deterioration of collagen synthesis in the blood vessels, and severe cough. For instance, it was stated that smoking inhibited directly the collagen synthesis. The role of smoking on anti-estrogenic effect might depend on its negative effect on collagen quality and its stimulating effect on smooth muscle tonus due to the change of the activity of the alpha-adrenergic receptors (Tampakoudis et al. 1995).

## Conclusion

Although urinary incontinence is a treatable condition, numerous women experience psychological, social, and physical problems due to this disorder. In this study, it was determined that 37 % of the women had UI and the rate of visiting a physician for this complaint was low. This result might be interpreted as an indicator that the women did not consider urinary incontinence as a serious health problem. Unfortunately, common misunderstandings about UI are continuing and therefore, treatment of UI in these women is delayed. Preventing of making UI tabooed, the perception concerning no need for embarrassment, and its treatment with simple methods like pelvic floor exercises and providing proper incentives for the treatment of the incontinence would be very useful for the development of the healthcare-seeking behavior. It is required for future studies to focus on in-depth research in order to understand the reasons for the avoidance of seeking healthcare service.

It was found that based on the correlation of UI with medical and obstetric characteristics and the logistic regression analysis of the risk factors; number of children, duration of delivery lasting longer than 24 h, and urogenital infections were significantly correlated with UI, but not the age at first and last childbirth, presence of episiotomy, childbirth with a birth weight over 4 kg, and smoking. In order to prevent UI affecting negatively the health and the quality of life of women, high multiparity, urinary infection, and diabetes should be addressed. Nurses and midwives have an important role in the education of women regarding prevention of the risk factors like obesity, constipation, urinary infections, and Kegel exercises. In conclusion, in order to eliminate the negative effects of UI on quality of life, it is essential to focus on prevention of UI’s risk factors.

## Abbreviations

UI: urinanry incontinence
POP: pelvic organ prolapse
